# An Observational Study on the Molecular Profiling of Primary Melanomas Reveals a Progression Dependence on Mitochondrial Activation

**DOI:** 10.3390/cancers13236066

**Published:** 2021-12-01

**Authors:** Jeovanis Gil, Melinda Rezeli, Elmar G. Lutz, Yonghyo Kim, Yutaka Sugihara, Johan Malm, Yevgeniy R. Semenov, Kun-Hsing Yu, Nga Nguyen, Guihong Wan, Lajos V. Kemény, Sarolta Kárpáti, István Balázs Németh, György Marko-Varga

**Affiliations:** 1 Division of Oncology, Department of Clinical Sciences, Lund University, 222 42 Lund, Sweden; yonghyo@krict.re.kr (Y.K.); yutaka.sugihara@med.lu.se (Y.S.); gyorgy.marko-varga@bme.lth.se (G.M.-V.); 2 Section for Clinical Chemistry, Department of Translational Medicine, Lund University, Skåne University Hospital Malmö, 205 02 Malmö, Sweden; johan.malm@med.lu.se; 3 Clinical Protein Science & Imaging, Biomedical Centre, Department of Biomedical Engineering, Lund University, 222 42 Lund, Sweden; melinda.rezeli@bme.lth.se; 4 Department of Dermatology, Venereology and Dermatooncology, Semmelweis University, 1085 Budapest, Hungary; elmar.lutz@stud.semmelweis.hu (E.G.L.); lkemeny@mgh.harvard.edu (L.V.K.); karpati.sarolta@med.semmelweis-univ.hu (S.K.); 5 Data Convergence Drug Research Center, Therapeutics and Biotechnology Division, Korea Research Institute of Chemical Technology (KRICT), Daejeon 34114, Korea; 6 Department of Dermatology, Massachusetts General Hospital, Harvard Medical School, Boston, MA 02110, USA; YSEMENOV@mgh.harvard.edu (Y.R.S.); NNGUYEN33@mgh.harvard.edu (N.N.); GWAN@mgh.harvard.edu (G.W.); 7 Department of Biomedical Informatics, Harvard Medical School, Boston, MA 02115, USA; Kun-Hsing_Yu@hms.harvard.edu; 8 Department of Dermatology and Allergology, University of Szeged, 6720 Szeged, Hungary; nemeth.istvan.balazs@med.u-szeged.hu; 9 Chemical Genomics Global Research Lab, Department of Biotechnology, College of Life Science and Biotechnology, Yonsei University, Seoul 03722, Korea; 10 1st Department of Surgery, Tokyo Medical University, Tokyo 160-8582, Japan

**Keywords:** primary melanoma, proteogenomics, PTMs, malignant melanoma, mitochondria, mitochondrial translation, lysine acetylation stoichiometry, MHC complex, antigen presentation, disease progression

## Abstract

**Simple Summary:**

Malignant melanoma is the deadliest among the skin cancers. In the advanced stage, it shows extremely poor prognosis. The incidence and mortality of melanoma has been continuously increasing worldwide, primarily among the fair-skinned populations. To date, the best indicator for progression is the tumor thickness (Breslow). In this sense, there is a fundamental need to discover early indicators of disease progression. We believe that in-depth molecular profiling at the multiomic level of primary melanomas may find a signature that predicts disease progression toward metastasis. Here we report an observational study of a small primary melanoma cohort, in which in-depth molecular profiling was performed at the levels of protein expression, phosphorylation, and lysine acetylation. Our results provided evidence for dysregulation of pathways and biological processes at all molecular levels evaluated, in primary tumors from patients that ultimately developed a metastatic disease. Moreover, we provided evidence at the molecular level of the sex-related differences in the melanoma disease presentation.

**Abstract:**

Melanoma in advanced stages is one of the most aggressive tumors and the deadliest of skin cancers. To date, the histopathological staging focuses on tumor thickness, and clinical staging is a major estimate of the clinical behavior of primary melanoma. Here we report on an observational study with in-depth molecular profiling at the protein level including post-translational modifications (PTMs) on eleven primary tumors from melanoma patients. Global proteomics, phosphoproteomics, and acetylomics were performed on each sample. We observed an up-regulation of key mitochondrial functions, including the mitochondrial translation machinery and the down-regulation of structural proteins involved in cell adhesion, the cytoskeleton organization, and epidermis development, which dictates the progression of the disease. Additionally, the PTM level pathways related to RNA processing and transport, as well as chromatin organization, were dysregulated in relation to the progression of melanoma. Most of the pathways dysregulated in this cohort were enriched in genes differentially expressed at the transcript level when similar groups are compared or metastasis to primary melanomas. At the genome level, we found significant differences in the mutation profiles between metastatic and primary melanomas. Our findings also highlighted sex-related differences in the molecular profiles. Remarkably, primary melanomas in women showed higher levels of antigen processing and presentation, and activation of the immune system response. Our results provide novel insights, relevant for developing personalized precision treatments for melanoma patients.

## 1. Introduction

Malignant melanoma, which develops from pigment-containing cells known as melanocytes, is among the most aggressive cancers and is the deadliest type of skin cancer. Both incidence and mortality are continually increasing, mainly among fair-skinned populations [1]. Skin exposure to environmental UV light, low levels of skin pigment, a large number of pigment nevi, genetic and environmental factors, and a compromised immune system are considered the most important factors in the development of the disease [2]. The main prognostic factor for melanoma, histopathological staging based on tumor features, has remained unaltered for several decades. It focuses primarily on tumor thickness, ulceration and metastases to lymph nodes or other organs; and it is a major estimate of the clinical behavior of primary melanoma.

Malignant melanoma is the most genetically heterogeneous cancer [3]. The high heterogeneity is evident at both inter- and intra-tumor levels, and this has been previously associated with metastatic progression of the disease with overall poor survival [4,5]. Using in-depth proteomics on a melanoma cohort, it was possible to determine the levels of mutated driver proteins. Specifically, the BRAF V600E mutated protein, which is the most frequent driver mutation in melanoma (accounting for ~50% of cases [6]), was correlated with dysregulation of functional signaling pathways with a resulting strong impact on tumor development and patient survival [7]. This highlights the relevance of deep molecular profiling to better understand the processes of development and progression of the disease, as well as, assessing better risk factors and predictors of therapeutic response.

In recent times, major upgrades in high-throughput molecular profiling of melanomas have been achieved, resulting in a broad characterization of the disease presentation [8,9]. With a special emphasis on the protein and post-translational modification (PTM) level, it is now possible to identify and quantify thousands of PTM events from minute amounts of starting material in melanoma tissue [10,11,12,13,14]. In particular, by integrating in-depth proteomics and PTMs, detailed histopathological characterization assisted by an AI algorithm and clinical information on 500 melanoma samples, our group presented the first blueprint of the Human Melanoma Proteome Atlas [15,16].

Here, we present our observations from in-depth high-throughput proteomics, phosphoproteomics, and acetylomics on a small cohort of primary melanomas composed of 11 patient tumors. The analysis was performed combining a workflow that allows simultaneously running quantitative proteomics, phosphoproteomics, and acetylomics on the same sample. From the molecular profiling we identified the activation of mitochondrial pathways such as the translation and organization in tumors from patients with disease progression. In addition, we observed differences at the transcript level in a large public dataset when comparing similar groups, and when comparing metastasis versus primary tumors. Moreover, we explored the differences in the mutational load between primary and metastatic melanomas. Our findings point to the mitochondria as a key player in melanoma progression. The exploration of the disease presentation between women and men, revealed differences related to immune system response, specifically in antigen processing and presentation. Altogether, our results highlight the importance of molecular profiling for finding predictors of disease progression, opening new opportunities for developing personalized precision treatment strategies.

## 2. Materials and Methods

### 2.1. Sample Collection and Processing

The 11 patients enrolled in the study turned with their primary melanoma of the skin to the Department of Dermatology, Venereology and Dermatooncology, Semmelweis University, Budapest, Hungary. They underwent surgery for primary excision. Native tumor tissue sampling was made by a dermatopathologist from the neighboring area of the thickest part of the tumor to assess the diagnosis of the patient. Tumors were snap-frozen and stored at −80 °C. Frozen tissue pieces were sliced for histological characterization and omic analysis at a fix 10 μm thickness, as previously described [14,16]. Briefly, 15 consecutive slices (10 μm thickness) were collected for proteomics and PTM analysis while the previous and following sections were placed on glass slides and stained with hematoxylin and eosin (H&E). Stained slides were scanned with an automated Aperio CS2 scanner (Leica Biosystems, San Diego, CA, USA). The tissue sample composition e.g., tumor cell, immune cell, and stroma content was determined based on the scanned images by the certified pathologist in our team assisted by the software QuPath [17] (v0.2.0-m8).

The patients were enrolled in the study when they were diagnosed with melanoma during May–October 2017. For progression toward a metastatic disease, the date of the first metastatic event and the type of metastasis was extracted from the clinical history of the patients. The information was last updated in May 2021.

### 2.2. Sample Preparation for Proteomics and PTM Analysis

Tissue sample slides were added a buffer for protein extraction (SDS 2%, DTT 50 mM, Tris 100 mM, pH 8.6), rest for one minute at 4 °C, and submitted to a sonication step on a Bioruptor following a setting of 40 cycles of 15 s On/Off at high intensity. The temperature was kept at 4 °C. For reduction and alkylation of disulfide groups, samples were first incubated at 95 °C for 5 min, and then added iodoacetamide 100 mM, incubated at room temperature in darkness for 20 min. Samples were immediately submitted to protein precipitation by adding 9 volumes of ethanol (−20 °C) overnight. Proteins dissolved in SDS 1%, sodium deoxycholate (SDC) 0.5%, TEAB 100 mM, were fully chemically acetylated their free amino groups by two consecutive additions of 100 molar excess of *N*-acetoxy-succinimide-d3 (NAS-d3) as previously reported [18]. Samples were treated with hydroxylamine 5% to revert *O*-acetylation. The excess of reagents and detergent were removed by protein precipitation as described above. Proteins dissolved in ammonium bicarbonate 50 mM, SDC 0.5% were digested with trypsin at a 1:50 enzyme:protein ratio. The reaction was incubated at 37 °C for 16 h. The peptide mixture was submitted to a SDC removal step by TFA acidified ethyl-acetate extraction [18], and stored at −80 °C until phosphopeptide enrichment or MS analysis.

### 2.3. Phosphopeptide Enrichment

A total of 80 μg of peptides was submitted to an automated workflow for the isolation of phosphopeptides on an AssayMAP Bravo system (Agilent Technologies, Singapore) as previously described [10]. Briefly, cartridges filled with 5 μL Fe(III)-NTA were used following the Phospho Enrichment protocol controlling the system. The peptides were previously dissolved in ACN:H_2_O (80:20), TFA 0.1% and loaded into pre-equilibrated cartridges at a flow rate of 3.5 μL/min. Phosphopeptides were eluted with ammonia 5%, collected directly into a formic acid solution, dried and stored at −80 °C until MS analysis.

### 2.4. Liquid Chromatography Mass Spectrometry Analysis

Peptides and phosphopeptide from individual samples or pools were spike with iRT and analyzed in a high-resolution LC-MS system (Ultimate 3000 RSLCnano UPLC (Dionex) coupled to a Q-Exactive HF-X mass spectrometer (Thermo Fischer Scientific, Waltham, MA, USA). Peptide samples were loaded and desalted on a trap column Acclaim PepMap100 C18 (3 µm, 100 Å, 75 µm i.d. × 2 cm, nanoViper) with aqueous 0.1% TFA at 5 μL/min for 6 min. The separation of peptides was performed by in-line, connecting the trap column with an EASY-spray RSLC C18 analytical column (2 µm, 100 Å, 75 µm i.d. × 50 cm) at a flow rate of 300 nL/min. The temperatures of the trap and analytical columns were set at 35 °C and 60 °C respectively. The peptides were separated following a non-linear gradient of 137 min, from 2–27% of B in 112 min, then from 27–35% and from 35–55% in 10 min each and finally to 90% in 5 min.

DDA analysis: to generate lysine acetylation raw files and the Peptide Spectral Libraries for global proteomics and phosphoproteomics, pools and individual samples were acquired following a data-dependent acquisition (DDA) method. The parameters to acquire the Phosphopeptide spectral library include a resolution of 120,000 (@ 200 *m/z*), an ACG target of 3 × 10^6^, and a maximum injection time of 50 ms for the MS1. The top 15 signal over the mass range of 385–1460 *m/z* are selected for MS2 at a resolution of 60,000 (@ 200 *m/z*), an ACG target of 1 × 10^5^, a maximum injection time of 120 ms, the isolation window of 1.2 *m/z*, a threshold of 8.6 × 10^2^ for ion selection, and the normalized collision energy (NCE) of 25. Selected ions were excluded for 30 s. For global Peptide Spectral library and lysine acetylation data acquisition the parameters for MS1 are as follows: resolution 120,000 (@ 200 *m/z*), ACG target 3 × 10^6^, maximum injection time 100 ms; the top 20 signals excluding single charge ions. MS2 of selected ions are acquired at a resolution of 15,000 (@ 200 *m/z*), ACG target 1E + 05, maximum injection time 50 ms, isolation window 1.2 *m/z*, minimum ACG target for ion selection 8 × 10^3^, and NCE 28. Selected ions were excluded for 40 s.

DIA analysis: the method for global proteomics and phosphoproteomics is designed for peptide/protein quantification in the MS1. A full acquisition cycle is composed of 54 variable width MS2 scans and 3 MS1 over the mass range of 385–1460 *m/z* distributed every 18 MS2 scans. The width of the MS2 windows were selected based on the distribution of signals identified during the acquisition of the Peptide Spectral Libraries, with an overlap of 1 Da between adjacent windows. The parameter for the MS1 were: resolution 120,000 (@ 200 *m/z*), ACG target 3 × 10^6^, and maximum injection time 50 ms; for MS2 the acquisition was at a resolution of 30,000, the ACG target of 1 × 10^6^, the maximum injection time was left in automatic, and the NCE was set at 28 and 25 for global proteomics and phosphoproteomics, respectively.

### 2.5. Peptide/Protein and PTMs Identification

Generation of Peptide Spectral Libraries: the raw data acquired in DDA mode (pools and individual samples) were analyzed with Spectronaut software (Biognosys, AG, Schlieren, Switzerland) using as a reference the human proteome database downloaded from UniProt in 2019. The parameters included selecting ArgC as the digestion enzyme, 2 missed cleavages allowed, fix modifications: carbamidomethyl cysteine, and acetylation with 3 deuterium (3d) in lysine, variable modifications: methionine oxidation, acetylation d0 and d3 in the N-terminal of proteins. The number of IDs was controlled by FDR of 1% at peptide and protein level.

Global proteomics: DIA runs were analyzed using Spectronaut. The generated Peptide Spectral Library, and similar parameters as for the library generation were used for peptide and protein identification. For proteome profiling a label free approach was selected based on peptide intensity in the MS1. Identified peptides and protein were controlled by FDR of 1% at both levels.

Phosphoproteomics: raw DDA data from pools of samples enriched in phosphopeptides were analyzed with Spectronaut software (Biognosys, AG) using the same search parameters as above mentioned. In addition, phosphorylation in residues of serine, threonine and tyrosine was included as variable modification. Only those phosphopeptide spectra with at least 75% confidence for the correct phosphorylation site localization were included in the library. DIA runs from samples under study were analyzed with Spectronaut using the phosphopeptide spectral library generated. Additionally, a direct DIA analysis was performed using the same criteria as for the search using spectral library. The quantification at phosphopeptide level was performed based on the intensity of the precursor ions in the MS1. Positive identifications were controlled by FDR of 1%.

Acetyl-lysine peptides identification and stoichiometry estimation: raw DDA data from global proteomics samples were analyzed with Pview software which allows the identification and stoichiometry calculation of acetylated peptides. The parameters for peptide/protein identification were the same as mentioned above for global proteomics spectral library building. For stoichiometry calculation the parameters were as previously reported [12,18]. Basically, a tolerance 3.5 ppm was allowed for the isotopic distribution and at least four peaks of the distribution must be registered to be considered for calculation.

### 2.6. Data Processing, Statistical and Functional Analysis

Protein and peptide abundance data from global proteomics and phosphoproteomics respectively were processed under Perseus platform [19]. Values were first log2 transformed and subtracted by the median intensity of all entries in the sample. Proteins with less than three valid values in the cohort were filtered out. In total, 9436 proteins were confidently identified and quantified including 7202 in more than 70% of the samples. The phosphoproteomics and acetylomics resulted in the quantification 12,114 and 4408 modified peptides respectively (Table S2).

The proteome and PTMs dysregulation between the progression status related groups (no progression *n* = 5, and progression *n* = 5) and sex (female *n* = 5, and male *n* = 6) was explored through an ANOVA test. The threshold for considering proteins and modified peptides as dysregulated between the groups was a *p*-value of 0.01. Functional enrichment analysis of differentially abundant proteins between groups was performed using the ToppCluster function of the online version of ToppGene Suite [20]. In addition, the medians of the protein abundances in each group were subtracted (progression—no progression and female—male), and the differences were used as the input for a 1D functional annotation enrichment analysis provided by the Perseus platform [21] using default parameters. The biological annotations of the proteins were taken from GO biological processes and KEGG pathways. A simple regression analysis between the Breslow thickness of the tumors and the protein profiles was performed and the results are presented in Table S2. Significantly positive or negative correlation of the proteins was considered using a threshold of *p*-value of 0.05.

For lysine acetylation stoichiometry analysis, abundance profiles of endogenous acetylated peptides (d0) were expressed as the percentage of the total distribution (sum of d0 and d3). For comparison between groups of samples, a probit transformation was applied to the values in percentage. The probit transformed data were submitted to 1D functional annotation enrichment analysis between the progression and sex groups as mentioned above. Only those acetylated peptides with at least two valid values in each group were considered for the analysis. A *p*-value of 0.05 was used for truncation of the biological annotations.

### 2.7. Transcriptomic Analysis

TCGA transcriptome data were obtained through the Firehose portal. Corresponding clinical data were obtained from cBioportal [22,23]. We used 472 samples that had corresponding transcriptomic and clinical data available and performed GSEA analyses [24,25]. We performed two GSEA analyses. In one approach we compared primary tumors (89) only and grouped them by progression status (progression and non-progression). Primary tumors that had “NA” for disease free status were excluded. In an alternative approach, primary and metastatic melanoma samples (total 472) were used for the GSEA. Both analyses ran using the “Gene Ontology Biological Process” gene set database at default GSEA settings with 1000 permutations. Significantly enriched pathways are based on a nominal *p*-value < 0.05.

## 3. Results and Discussion

### 3.1. Histopathological Characterization of the Cohort

According to the WHO classification, malignant melanoma is subtyped according to the site, degree of ultraviolet sun exposure, and the type of histological spreading. The two most common subtypes include superficial spreading melanoma (SSM), with or without vertical spreading, and nodular melanoma (NM), which has a unique vertical growth pattern. Vertical growth is considered even in the superficial spreading cases if tumorous nests invade at least Clark level III and/or solid areas filling the entire dermal papilla. Since the absolute thickness (Breslow) is the main prognostic indicator for the clinical behavior of melanomas, the presence of a vertical growth phase is associated with a worse prognosis. However, within a range of thickness there is no widely used histological prognostic indicator for individual cases.

**Figure 1:**
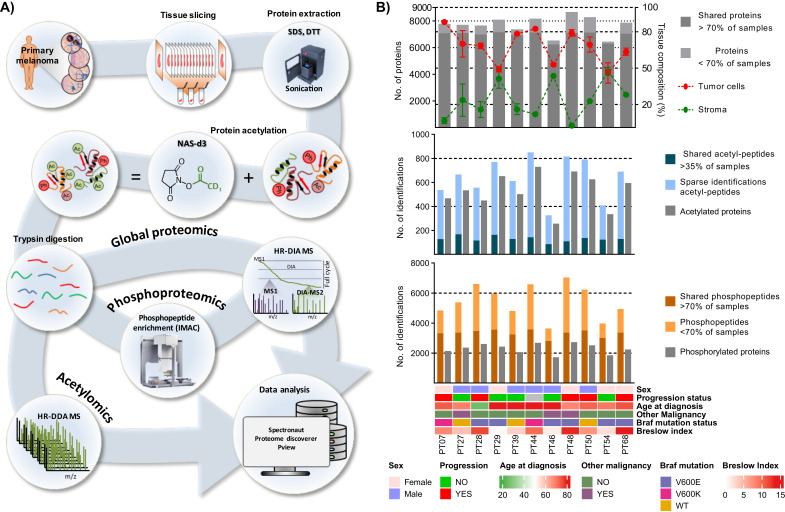
Multiomic analysis of primary melanomas. (**A**) Sample preparation workflow for global proteomics, phosphoproteomics and acetylomics. (**B**) Distribution of proteins identified in the primary melanoma cohort and the tissue composition according to the histopathological characterization (% of tumor cells and stromal compartment) (top panel), distribution of the PTMs identified (acetylation and phosphorylation) (middle and bottom panels), and relevant clinical data of the patients and samples in the cohort study.

**Figure 2:**
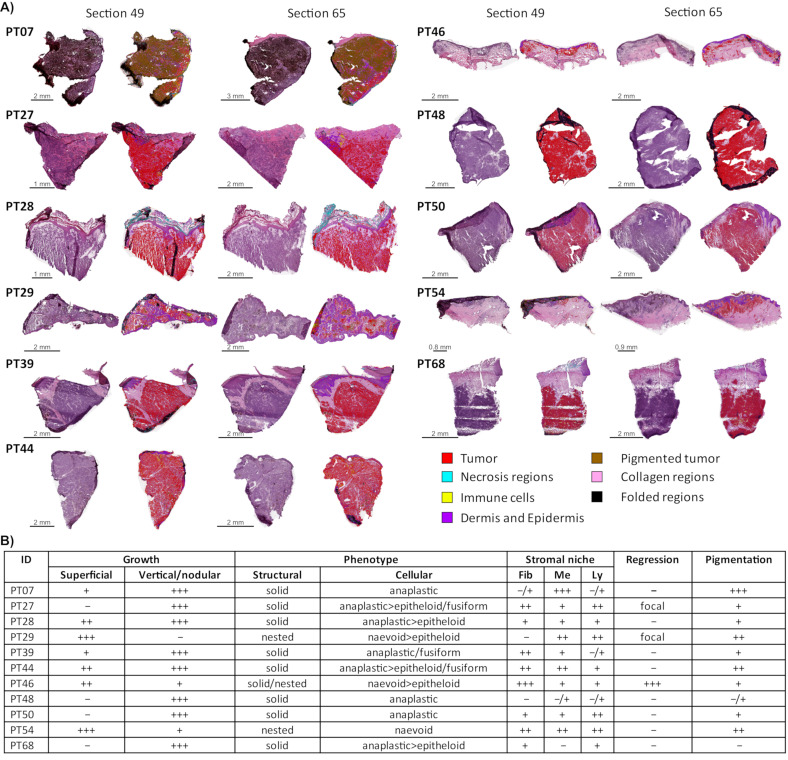
Histopathological characterization of the cohort of primary melanomas. (**A**) Tissue sections before and after the scanner (Leica Biosystems, San Diego, CA, USA), and the digital images were submitted to histopathological characterization of the tissue composition. Histological images were analyzed under the QuPath platform using a cell-based profiling mode. Each section is presented as a H&E scanned image (left) and an annotated image as the output of the analysis platform (right). The summary of the histological characterization is outlined in Table S1. All images are arranged as top-down (from skin surfaces to inside of skin). (**B**) Representation of the histopathological characterization of the examined primary melanoma samples according to standardized dataset involving the growth, structural and cellular phenotypes, stromal niche variables (Fib: fibrotic/desmoplastic reaction; Me: melanophages; Ly: lymphocytic infiltration), regression, as well as pigmentation. For the extension of the horizontal spreading of the melanoma ‘+’ indicates focal superficial spreading counterpart which limited less than 25% of the total horizontal extension of the examined tumor sample without the dominancy of the superficial spreading component. The ‘++’ indicates well recognizable superficial counterpart occupying >25% of the total horizontal extension; however, with a dominant vertical spreading/regressive area. The ‘+++’ indicates a marked and dominant superficial spreading component >75% of the total horizontal extension of the examined tumor sample. Indeed ‘+++’ in the regression column indicates a dominant diffuse (>75%) regressive component within the tumor sample (PT46). Similarly, intratumoral pigmentation was semiquantitatively counted by ‘+++’ diffuse and marked pigmentation, ‘++’ scattered focal, but pronounced pigment accumulation, ‘+’ focal and moderate intratumoral pigmentation, ‘−/+’ focal mild pigmentation within the tumor cells.

A cohort composed of eleven primary melanomas surgical isolated and stored as fresh-frozen (−80 °C), was selected for multiomics analysis following a sample preparation workflow that allows, from the same starting material, to analyze both global proteomics, phosphoproteomics, as well as acetylomics (Figure 1A). Dissected melanoma tumors were sliced for hematoxylin and eosin (HE) staining and multiomic analysis was performed, as previously reported [14]. Stained tissue sections delimit those used for multiomics and both were considered for computing the sample composition. Summary of the tissue composition determination of each histological image, and the raw and QuPath processed images are presented in Table S1 and Figure 2A. In particular, tumor cell and stromal content in the tissue sections were plotted in Figure 1B.

The selected samples for deep multiomics were all pathologically characterized. We processed the histopathological parameters by semiquantitative assessments, according to the routinely used histopathological schemes (Figure 2B). The entities are in-line with the WHO classification recommendations, as well as, evaluations of the regression published by our institutional study [26]. All primary tumors submitted to the study showed a vertical growth phase with focal superficial growth (Figure 2B). In four samples, the total vertical growth dominated (PT27, PT48, PT50 and PT68) whereas in two samples the superficial spread dominated the picture (PT29 and PT54). Within the mainly solid areas, anaplastic cells and focal epitheloid change were noted, the naevoid structures formed a nested pattern. In three cases, a sarcomatous phenotype was noted as well. The stromal niche varied among the samples from a stroma-dominant regressive stroma to the pauci-cellular microenvironment. The tissues were mainly pigmented, to a variable degree. In the heavily pigmented tumor tissues, both the melanophages and the tumor cells contained abundant melanosomes. One case showed massive regression, with fibrosis and lymphohistiocytic infiltration (PT46); two more cases exhibited focal regressive areas (PT27 and PT29). There was no massive lymphocytic permeation noted. A summary of the characterization is reported in Figure 2B.

### 3.2. Multiomics Analysis of Primary Melanoma Specimens

Sample preparation procedures for multiomics include a protein extraction step in high SDS content assisted by sonication and chemical acetylation (carrying heavy isotopes d3) of all lysine residues [18]. Fully acetylated proteins were digested with trypsin, generating peptides delimited by arginine residues. A fraction of the peptide sample was submitted to a phosphopeptide enrichment step assisted by a robotic liquid handling system [10]. For global proteomics and phosphoproteomics, samples were analyzed by high-resolution mass spectrometry following a Data Independent Acquisition (DIA) method, designed for a reliable peptide and protein quantification in the MS1. Variable width MS2 windows were determined considering the empirical *m/z* distribution of the peptides under similar analytical conditions (see details in the Experimental Section). For acetylomics, data were acquired in Data-Dependent Acquisition (DDA) mode (Figure 1A). A high-resolution mass spectrometer (Q-Exactive HFX) was used for all measurements.

The global proteomic output provided quantitative data on 9436 different proteins, ranging from 6443 to 8663 in individual samples. A total of 7202 proteins were confidently quantified in more than 70% of the samples (Figure 1B). At the PTM level, from the acetylomic analysis we were able to determine the site-specific occupancy of acetylation on lysine residues on 4408 peptides corresponding to 2514 different proteins. The distribution of identified acetylated peptides and proteins across the sample cohort is illustrated in Figure 1B. The phosphoproteomics resulted in the identification/quantification of 12,114 phosphorylated peptides corresponding to 3587 phosphoproteins. The numbers of identifications in each sample are presented in Figure 1B. The clinical data of the patients and tumors in the cohort is summarized in Table S1. Relevant patient information such as sex, age at diagnosis, the presence of a malignancy other than melanoma, and the progression status of the melanoma, as well as the BRAF mutation status and the Breslow thickness of the primary tumors are shown in Figure 1B. For further analysis, samples were grouped based on the progression status of the disease toward metastatic: No progression (*n* = 5), Progression (*n* = 5), and No Information (*n* = 1); and based on the sex of the patients: Female (*n* = 5), and Male (*n* = 6). Samples in the No progression group belong to patients without signs of metastatic events related to their primary melanoma during the period between the primary excision in 2017 and May 2021. For patients in the Progression group, the date and type of the first metastatic event are listed in Table S1. The time elapsed between the primary excision and the onset of metastasis ranged between 4 and 16 months. Of the five patients included in this group, two developed lymph node metastases, one subcutaneous metastasis, one lung metastasis, and the last one developed multiple metastases including subcutaneous, lung and liver metastases (Table S1).

### 3.3. Functional Analysis of Melanoma Progression Status

**Figure 3:**
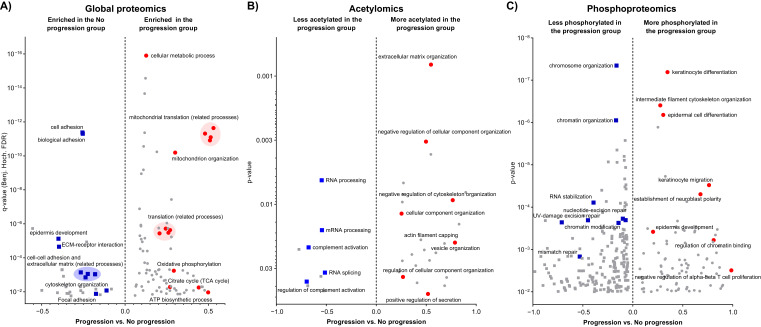
Functional annotation enrichment analysis of the difference at the proteome level (**A**) at the acetylome (**B**), and at the phosphoproteome level (**C**), between the groups of primary melanomas from patients where the disease progressed and those without metastatic event. Highlighted in red and blue are relevant annotations enriched in the Progression and No progression groups respectively.

The three datasets corresponding to the global proteomics and PTM analyzes were consulted to investigate whether we can find a molecular profile signature for progression to metastatic disease in primary melanomas. The differences between the average abundance profiles of individual entries (proteins or PTMs) in the progression and no progression groups were used for 1D functional annotation enrichment analysis [21]. At the proteome level, the group where the disease progressed, was significantly enriched in the mitochondrial translation machinery and, to a lesser extent, the TCA cycle, and the oxidative phosphorylation. This was also proven to be the case for the mitochondrial organization process within the same group of primary melanomas. On the contrary, biological functions such as cell adhesion, extracellular matrix organization, and epidermis development were significantly underrepresented in the progression group (Figure 3).

At the PTMs level, the progression group presented higher acetylation in proteins involved in the organization of the extracellular matrix, cellular components, cytoskeleton, and vesicles. Oppositely, proteins involved in RNA processing and splicing exhibited less acetylation occupancy in their lysine residues (Figure 3). On the other hand, the phosphoproteome analysis revealed a dysregulation between the progression groups in critical pathways and processes related to tumor development and progression. In particular, we found under-phosphorylated processes linked to DNA damage repair and chromatin organization in the progression group. In addition, processes that were found down-regulated at the proteome level such as the epidermis development, were differentially regulated by phosphorylation, suggesting a potential mechanism for the dedifferentiation (Figure 3).

**Figure 4:**
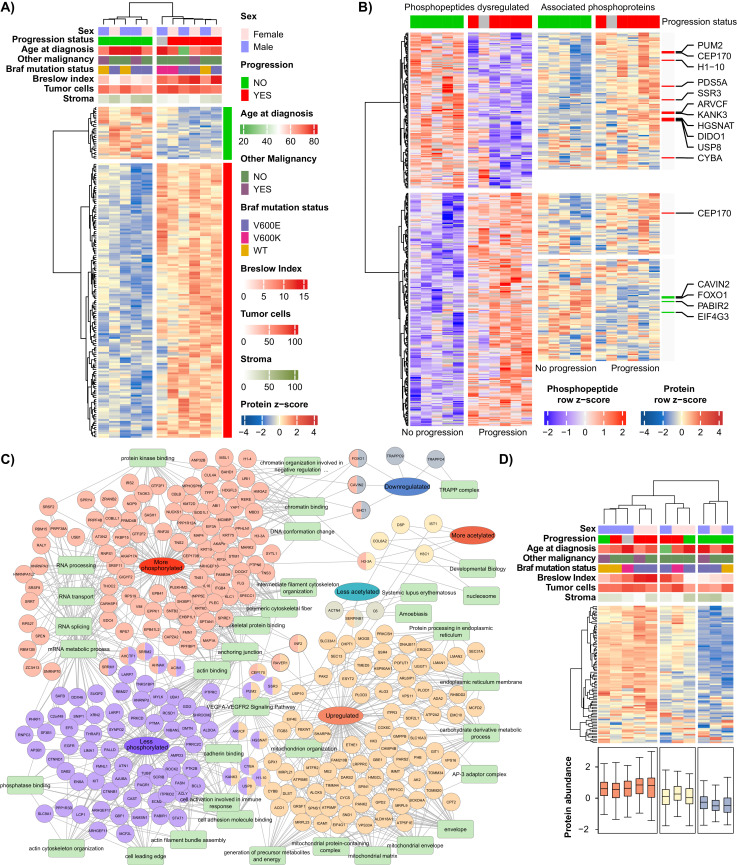
Molecular signature of the progression of primary melanomas toward metastatic disease. (**A**) Heatmap illustration of the significantly dysregulated proteins in primary melanomas from patient where the disease progressed. (**B**) Heatmap of the phosphorylated peptides dysregulated between the progression status groups, and their corresponding phosphoproteins. (**C**) Protein-biological annotation network of the clusters of proteins from differential abundance analysis of the proteins and PTMs dataset related to the progression of the disease. The analysis was performed using the ToppCluster function of the ToppGene Suite online version [20]. (**D**) Clustering analysis and heatmap illustration of the protein elements of the mitochondrial translation machinery. The corresponding abundance of the proteins in each sample is represented as a boxplot graph.

A more specific analysis, considering the proteins and PTMs significantly dysregulated between the progression status groups (ANOVA, *p*-value < 0.01) revealed similar results. The comparison at the proteome level between the abundance profiles of the progression status groups revealed that 175 proteins were significantly dysregulated, 28 down- and 147 up-regulated in the progression group (Figure 4A, Table S2). On the other hand, the differential abundance analysis of phosphorylated peptides resulted in the identification of 372 dysregulated phosphopeptides, 149 down- and 233 up-regulated. Interestingly, the negative regulation of phosphorylation events in the progression group correlated with relatively higher levels of their corresponding phosphoproteins, including 11 where the up-regulation was statistically significant according to our criteria (Figure 4B). Oppositely, the relative profiles of phosphoproteins corresponding to their hyper-phosphorylated peptides clustered in two groups, one with relatively higher and the other with lower abundance levels in the progression group, including four significantly down-regulated proteins (Figure 4B). The acetylomics resulted in only 13 acetylated peptides significantly dysregulated between the progression status groups.

The six clusters of proteins corresponding to dysregulated elements of the three omic datasets were submitted to a functional enrichment analysis using the ToppCluster function of the ToppGene Suite online version [20]. The functional network is illustrated in Figure 4C. In the cluster of up-regulated proteins in the progression group, several mitochondrial functional annotations were significantly enriched, including mitochondrion organization and envelope, as well as, the generation of precursors metabolites and energy, suggesting a mitochondrial activation dependence for the melanoma progression. The VEGFA-VEGFR2 signaling pathway, which is involved in activating angiogenesis by inducing proliferation of endothelial cells [27], was found significantly up-regulated at the proteome level. Interestingly, the same pathway was enriched in the down-regulated phosphoproteome. A closer look at these phosphoproteins revealed that 6 out of the 12 hippo-phosphorylated proteins (PTK2B, PRKCD, STAT1, ROCK2, CTNNB1, and CTNND1) are also part of the signaling cascade driven by other receptor tyrosine kinases. This suggests that the regulation of these pathways plays a key role in melanoma progression and warns that the potential use of tyrosine kinase inhibitors in melanoma should be carefully investigated. The clusters of proteins dysregulated at the PTM level, particularly by phosphorylation, are involved in several groups of pathways, one linked to cytoskeleton organization, one to RNA-related processing, and another associated with DNA and chromatin organization. The first two pathway groups were found regulated by both under- and over-phosphorylation, while in the last one the progression correlates with up-regulation of the phosphorylation.

### 3.4. The Mitochondrial Translation Machinery as a Risk Factor for Melanoma Progression

The mitochondrial translation-related processes were significantly enriched at the proteome level in the progression group as mentioned above (Figure 3). Here we filtered the proteins involved in these processes to investigate their power to discriminate between the progression groups. A hierarchical clustering analysis was performed using the relative levels of these proteins. Interestingly, the sole levels of the 90 mitochondrial translational proteins identified in this study, discriminate patient samples into three groups (Figure 4D). The first group with the overall highest abundance of these proteins includes patients where a metastatic event was reported. In the same group, only one patient did not report any progression of the melanoma; however, shortly afterwards this patient was diagnosed with another malignancy, lung adenocarcinoma, and received the corresponding anticancer therapy. This may have prevented the progression of melanoma. Moreover, the patient without follow-up records also fit into this group with the highest levels of mitochondrial translation-related proteins. The second group, with medium abundance contains samples from three patients, including two from the progression group, suggesting that small up-regulation in this set of proteins may indicate a high-risk factor for melanoma progression. Finally, the group of samples with the lowest levels of the mitochondrial translation all belong to the no progression group (Figure 4D). Our results indicate a positive correlation between the levels of mitochondrial translation and the progression toward metastasis of primary melanoma. Moreover, the up-regulation of other mitochondrial pathways suggests a melanoma dependence on mitochondrial function. In this sense, we envision that specific mitochondrial inhibitors particularly mitochondrial ribosome inhibitors such as antibiotics of the tetracycline and macrolide families, or others, represent a potential therapeutic opportunity to treat high-risk melanomas. Our unpublished results (manuscript in preparation), and others have proposed the use of OXPHOS inhibitors and antibiotics to target cancer cells [28,29,30].

### 3.5. The Relation between the Tumor Thickness (Breslow) and the Progression of Primary Melanomas

**Figure 5:**
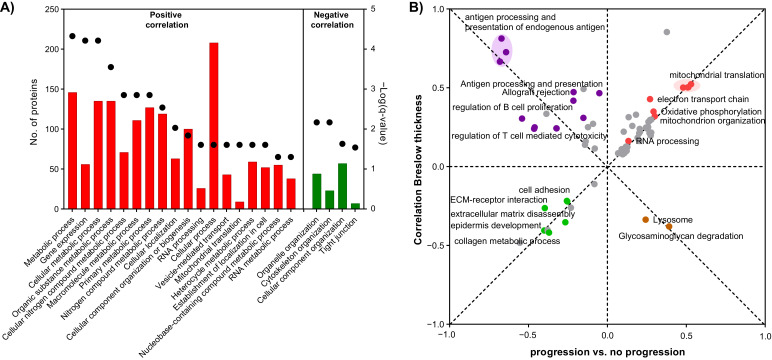
Functional biological annotations associated with the Breslow thickness of primary melanomas. (**A**) Functional enrichment analysis of the proteins with a significant correlation to the Breslow thickness of the tumors. (**B**) 2D functional enrichment analysis between the correlation at the proteome level with Breslow thickness of the tumors and the progression status of the patients. Highlighted in red are relevant annotations up-regulated in the progression group and with positive correlation with the Breslow thickness; in green, annotations down-regulated in the progression group and with negative correlation with Breslow thickness; in purple and brown are annotations oppositely correlate with progression and Breslow thickness.

In melanoma, for several decades the tumor thickness has been considered a major indicator for the disease progression [31]. In our cohort, we observed a clear difference in Breslow thickness (*p*-value < 0.01, Mann Whitney test) of tumors from the progression groups and from the no progression group, where the thicker tumors belong to the progression group (Figure 4A). We explored the interconnection between the Breslow thickness and the disease progression through proteome profiling. A simple regression analysis was performed between the Breslow thickness and protein levels (Table S2). As a result, a total of 377 proteins showed a significant correlation to the Breslow thickness (*p*-value < 0.05), 250 positive and 127 negative. The biological processes significantly enriched in both protein sets are represented in Figure 5A. Mostly metabolic and RNA-related processes were enriched among the proteins with positive correlations to Breslow thickness. Remarkably, the mitochondrial translation was found significantly enriched with nine protein elements. Oppositely, among the proteins with negative correlation with the Breslow thickness only the cytoskeleton organization and tight junction were significantly enriched.

The relation between Breslow thickness and the disease progression was explored through a 2D functional annotation enrichment analysis [21]. On the Breslow side we used the slope of the regression analysis, and on the progression side we used the difference between the average abundance of the proteins in the progression group (Figure 5B). The biological processes and KEGG pathways were used as the functional annotations of the proteins. As expected, several pathways showed significant correlations to both variables in the same direction. The mitochondrial translation, oxidative phosphorylation, and mitochondrial organization-related processes, as well as RNA processing, were overrepresented in thicker tumors and in the progression group, and tissue-specific pathways including the extracellular matrix organization were underrepresented. Remarkably, we identified pathways dysregulated in opposite direction between the two variables. Our analysis indicates that antigen processing and presentation together with pathways related to activation to the immune system are significantly underrepresented in the group of progression; however, they correlate with thicker tumors. In this sense, our observation suggests that the thicker tumors at a certain point activated the immune system response by antigen presentation.

### 3.6. Genomic Analysis on Melanoma Progression

**Figure 6:**
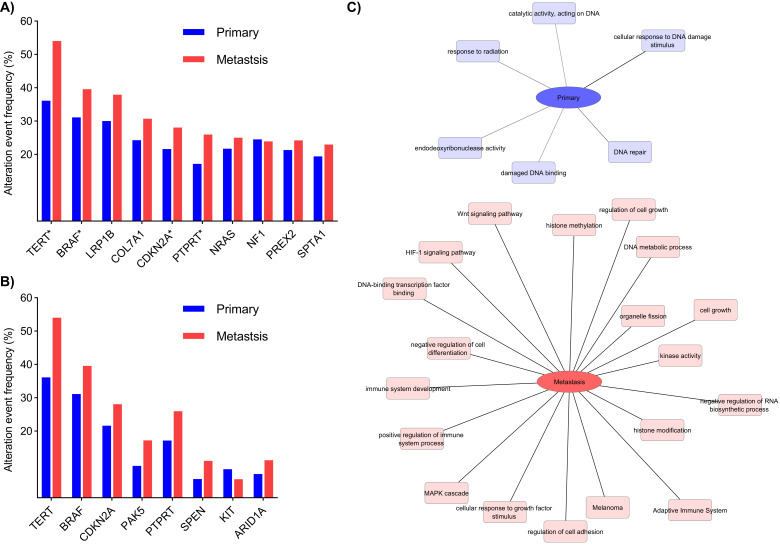
Most frequently mutated genes in primary and metastatic melanoma are involved in key pathways for the development and progression of the disease. (**A**) Genes with the highest frequency alterations among primary and metastatic tumor samples using the project GENIE melanoma dataset. * Denotes significant differences (*p* < 0.05) in the frequency of alterations between primary and metastatic tumor samples. (**B**) Genes with significant differences in frequency alterations among primary and metastatic tumor samples (*q*-value < 0.05). (**C**) Network representation of the functional clustering analysis of genes with differences in frequency alterations between primary and metastatic melanomas. The genes were filtered based on a *p*-value 0.05 and the analysis was performed using the ToppCluster function of the ToppGene Suite online version [20].

Project GENIE (Genomics Evidence Neoplasia Information Exchange) is an international cancer registry with genomic sequencing data [32]. To further investigate the melanoma progression, we looked for whether there are differences in mutational load in melanoma related to the progression toward a metastatic disease using the dataset from Project GENIE. The dataset consisted of 4547 melanoma tumor samples from 17 academic institutions within the United States. We stratified the tumor samples, as a proxy of progression, into primary and metastatic melanoma samples (1606 primary, 2941 metastasis) and compared the frequency of gene mutations using a two-sided, two-sample *t* test. *p*-values less than 0.05 after multiple testing adjustment were considered statistically significant. All analyses were completed in the cBioPortal platform [22,23] and summarized in Table S3. The most frequently mutated genes among the cohort are detailed in Figure 6A. The results indicate that among the most frequently altered genes, there are significant differences in the occurrence of mutations in the TERT, BRAF, CDKN2A, and PTPRT genes with enrichment in metastatic lesions (*p* < 0.01). In addition, there are significant differences in the occurrence of alterations in the PAK5, SPEN, KIT, and ARIDA1A genes (Figure 6B). A functional annotation analysis revealed that differentially mutated genes associated with progression are related to the regulation of signaling and transcription pathways affecting cellular growth and division, as well as immune system development (Figure 6C). Interestingly, the relatively more frequently mutated genes in primary melanomas are involved in DNA repair-related processes, suggesting that affecting the repair mechanisms is more relevant for melanoma development than for progression.

### 3.7. Transcriptomic Analysis on the Progression of Melanoma

**Figure 7:**
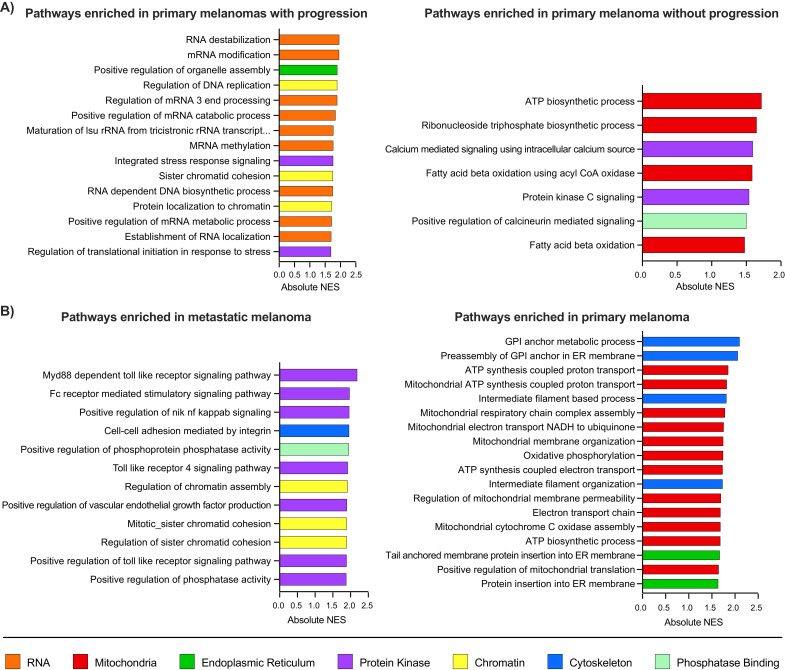
Transcriptionally dysregulated biological pathways associated with progression in melanoma. Gene set enrichment analysis (GSEA) using the GO: Biological Process gene set database showed significant enrichment of pathways associated with progression (**A**, left) and non-progression (right) in primary tumors from TCGA. Enriched Pathways in Melanoma Metastases vs. Primary tumors from TCGA (**B**). GSEA using the GO:Biological Process gene set database showed significant enrichment of pathways associated with metastases (left) and primary tumors (right).

Next, we aimed to investigate whether the pathways associated with progression on the proteomic and PTM level could be seen on the transcriptome level using a large cohort of melanoma samples from TCGA [8]. We have seen that patients with primary melanomas who progressed compared with patients who did not show progression were enriched in the pathways (Figure 7A, Table S4) that were previously identified using the proteomic datasets. Similarly, as a proxy of progression, we compared metastatic melanoma samples with primary melanomas and have found that similar pathways are differentially expressed (Figure 7B, Table S4). This suggests that the proteomic analysis described above identified pathways that may be recapitulated in larger transcriptomic datasets.

### 3.8. The Molecular Disease Presentation in Women and Men

**Figure 8:**
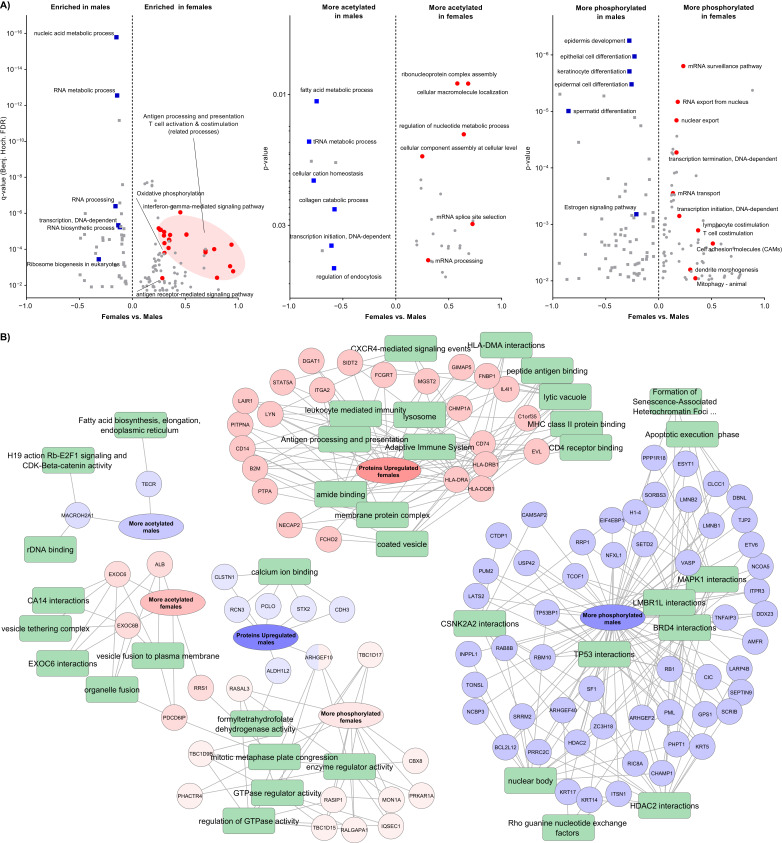
The sex-related differences in the melanoma proteome. (**A**) 1D functional annotation enrichment analysis of the differences at the proteome (left), acetylome (middle), and phosphoproteome (right) levels between women and men. (**B**) Functional annotation-protein network of identified elements dysregulated between women and men in the three datasets. the analysis was performed using the on the ToppCluster function of the ToppGene Suite online version [20].

Melanoma disease displays differences in both incidence and mortality between men and women. The disease is more frequent and more lethal in men according to the global statistics [1]. Recent findings however suggest a role for female sex hormone estrogen in the development and response to therapy in melanoma [33,34]. Here we performed functional annotation enrichment analyses on the three omic datasets to explore the differences in the disease presentation between women and men (Figure 8A). At the proteome level, we found processes related to the antigen processing and presentation including the MHC complexes, as well as pathways involved in the activation of the immune system response, shifted toward higher levels in women. Conversely, the analysis showed relatively higher levels of biological annotations related to ribosomal RNA processing, ribosome biogenesis, and transcription in tumors from men.

Similar analysis, by taking lysine acetylation occupancy into account, suggests a difference in the regulation of biological pathways and processes related to transcription and RNA processing. In women, higher acetylation was observed on proteins involved in the ribonucleoprotein complex and cellular component assembly. Correspondingly, within males, the transcription initiation related proteins were more acetylated, involved in the metabolism of fatty acids (Figure 8A). Noteworthy, due to the lower level of coverage of the acetylome in this study, confirmatory analysis should be conducted including a large cohort of samples. The acetylation stoichiometry provides relevant information on the potential use of anticancer drugs targeting the modulators or the targets of this PTM, such as HDAC inhibitors in melanoma. At the phosphoproteome level, the tissue-specific-related processes such as epidermis cell-, keratinocyte-, and epithelial cell differentiation were found more phosphorylated in men. In women, the more phosphorylated proteins are significantly enriched in transcription events and RNA processing and transport. Processes related to lymphocyte and T cell costimulation were more phosphorylated in women.

These findings provided insights into a disease presentation that partially could explain the sex-related differences for both incidences and mortality. The down-regulation of antigen processing and presentation in tumors from men contribute to evade the immune surveillance, could be stipulated. While on the other hand, we observed an up-regulation of proliferation related pathways, such as the biogenesis of the ribosomes.

The corresponding proteins of the elements significantly dysregulated between women and men in the proteomics, phosphoproteomics, and acetylomics datasets (ANOVA, *p*-value < 0.01), were submitted to a functional enrichment analysis (Figure 8B). The results indicate that up-regulated proteins in women are mainly involved in antigen processing and presentation, and interaction with the immune system. Among these proteins there are four elements of the MHC protein complex. It relates to three proteins from class I (B2M, HLA-B, HLA-E) and one from class II (HLA-DQB1) in addition to the PSMB8 (Proteasome subunit beta type-8) which is involved in the antigen processing for MHC class I. Conversely, among the up-regulated proteins in men only calcium ion binding was enriched. At the PTM level, in women more acetylation was identified in processes related to the fusion of organelles and vesicles to plasma membrane, while the regulation of GTPase activity was more phosphorylated. In men mainly nuclear proteins, known interactors of chromatin modulator and kinases such as MAPK1 and CSNK2A2, were more phosphorylated. Taken all together, our results provide evidence at a molecular level, of the differences in melanoma disease presentation between women and men. This suggests that the development of personalized treatment strategy must take sex into account.

## 4. Conclusions

This observational study on a small cohort of primary melanomas at the protein level, including phosphorylation and acetylation events, highlights the relevance of deep molecular characterization, as a way to understand the mechanism of the disease presentation. The cohort was suitable for investigating whether is possible find an early molecular signature, indicative of disease progression toward metastasis. It was remarkable to observed up-regulation of the mitochondrial translation machinery in primary tumors from patients where the disease progressed afterwards. Moreover, approximately 50% of the proteins significantly up-regulated in this group are involved in mitochondrial function, strongly suggesting a critical driving role of this organelle in melanoma progression. A similar mitochondrial pathways activation was observed in correlation to the Breslow thickness of the tumors. However, the tumor thickness positively correlated with functional annotations related to the immune system response including the antigen processing and presentation via MHC complex, which negatively correlated with progression of the disease. In addition, our results together with the analysis at the transcriptome and genome level, in large datasets on melanoma cohorts, indicate that each molecular level, contribute uniquely to the molecular signature of the disease progression.

Our in-depth molecular profiling in melanoma revealed sex-related differences associated with the interaction between the tumor and the immune system. Previous studies highlighted the differences in the response to immune check point inhibitors (ICIs) between men and woman in melanoma and other cancers [35]. There is a general tendency for men to respond better to ICIs in melanoma, as shown by clinical trials [36,37,38]. The relatively higher levels of the antigen processing and presentation, as well as other proteins involved in the activation of the immune response in women, support the hypothesis of low antigenicity of these tumors, which limits the ability to respond to ICIs [39]. In this sense, a combination of immunotherapy with a treatment that enhance the antigenicity of the tumors may be a potentially better therapeutic strategy.

Overall, our study highlights the importance of integrating data from different molecular levels to develop better prognostic factors, identify the vulnerabilities of the disease, select a better treatment strategy, and represents step forward to introduce true personalized medicine.

## Data Availability

The data reported is in the manuscript or as a Supplementary Materials linked to this publication.

## Supplementary Materials

The following are available online at https://www.mdpi.com/article/10.3390/cancers13236066/s1, Table S1: Summary of the clinical data of the patients and samples, tissue composition analysis, and histopathological characterization of the samples, Table S2: Multiomic quantitative data on the cohort of primary melanomas, Table S3: Summary of the genomic analysis when comparing metastasis and primary melanomas, Table S4: Summary of the GSEA at the transcriptome level when comparing primary tumors between progression status groups, and when comparing metastasis and primary melanomas.
